# Meta-analysis of surgical resection and radiofrequency ablation for early hepatocellular carcinoma

**DOI:** 10.1186/1477-7819-10-163

**Published:** 2012-08-16

**Authors:** Gang Xu, Fu-zhen Qi, Jian-huai Zhang, Guo-feng Cheng, Yong Cai, Yi Miao

**Affiliations:** 1 Department of General Surgery, Huai’an First People’s Hospital, Nanjing Medical University, 6 Beijing Road West, 223300, Huai’an, Jiangsu Province, People’s Republic of China; 2 Department of General Surgery, First Affiliated Hospital, Nanjing Medical University, 300 Guangzhou Road, 210029, Nanjing, People’s Republic of China

**Keywords:** Hepatocellular carcinoma, meta-analysis, radiofrequency ablation, surgery

## Abstract

**Background:**

There is no definite agreement on the better therapy (radiofrequency ablation (RFA) versus surgical resection (SR)) for early hepatocellular carcinoma (HCC) eligible for surgical treatments. The purpose of this study is to evaluate the evidence using meta-analytical techniques.

**Methods:**

A literature search was undertaken until December 2011 to identify comparative studies evaluating survival rates, recurrence rates, and complications. Pooled odds ratios (OR) and 95% confidence intervals (95% CI) were calculated with either the fixed or random effect model.

**Results:**

Thirteen articles, comprising two randomized controlled trials(RCTs), were included in the review, with a total of 2,535 patients (1,233 treated with SR and 1,302 with RFA). The overall survival rates were significantly higher in patients treated with SR than RFA after1, 3, and 5 years (respectively: OR, 0.60 (95% CI, 0.42 to 0.86); OR, 0.49 (95% CI, 0.36 to 0.65); OR, 0.60 (95% CI, 0.43 to 0.84)). In the SR group, the 1, 3, and 5 years recurrence rates were significantly lower than the RFA group (respectively: OR, 1.48 (95% CI, 1.05 to 2.08); OR, 1.76 (95% CI, 1.49 to 2.08); OR, 1.68 (95% CI, 1.21 to 2.34)). However, local recurrence between two groups did not exhibit significant difference. For HCC ≤ 3 cm in diameter, SR was better than RFA at the 1, 3, and 5 years overall survival rates (respectively: OR, 0.34 (95% CI, 0.13 to 0.89); OR, 0.56 (95% CI, 0.37 to 0.84); OR, 0.44 (95% CI, 0.31 to 0.62)). This meta-analysis indicated that the complication of SR was higher than RFA (OR, 6.25 (95%CI, 3.12 to 12.52); *P =* 0.000).

**Conclusion:**

Although local recurrence between two groups did not exhibit significant difference, SR demonstrated significantly improved survival benefits and lower complications for patients with early HCC, especially for HCC ≤ 3 cm in diameter. These findings should be interpreted carefully, owing to the lower level of evidence.

## Review

### Background

Hepatocellular carcinoma (HCC) is the seventh most common malignant tumor and the third leading cause of cancer-related deaths worldwide, with an estimated 500,000 deaths per year [1-3]. In past decades, developments of medical devices and interventional techniques have resulted in substantial opportunities for HCC early diagnosis and therapy.

Current options for the treatment of the early HCC conforming to the Milan criteria (single HCC ≤ 5 cm or up to three nodules ≤ 3 cm), that is stage I, consist of liver transplantation, surgical resection, transcatheter arterial chemoembolization (TACE), and percutaneous tumor ablation [4-7]. Theoretically, the best treatment is liver transplantation [8-13]. However, the limited availability of suitable living donors, as well as an increased waiting period, has raised the demand for treatment strategies of early HCC, such as SR and local ablation therapies. Comparison of different local ablative methods has shown that RFA is the most effective in terms of both morbidity and the elimination of tumors locally [14,15].

Some disputes, however, are reported about RFA and SR. Huang *et al.*[16], Molinari *et al.*[17], and Takayama *et al.*[18] reported that SR had more advantages (survival and recurrence rates) regardless of tumor size (larger or smaller than 3 cm; even smaller than 2 cm). However,Chen *et al.*[19], Hong *et al.*[20], Vivarelli *et al.*[21], and Montorsi *et al*. [22] concluded that RFA was as effective as SR in the treatment of solitary and small HCC. Additionally, Livraghi *et al*. [23] and Nashikawa *et al*. [24] considered RFA the first-line treatment for small resectable HCCs.

Whether RFA or SR is the better treatment for early HCC has long been debated. The aim of this review was to examine survival and recurrence rates after RFA and SR for HCC over the past decade.

## Materials and methods

### Literature search

Electronic searches were accomplished of the MEDLINE, Cochrane Controlled Trial Register (CENTRAL) and EMBASE databases until December 2011. The following MeSH search headings, all in English, were used: surgical resection, hepatic resection or hepatectomy; radiofrequency, radio-frequency or catheter ablation; and liver cancer or hepatocellular carcinoma.

### Data extraction and quality assessment

Two reviewers (Gang Xu and Fuzhen Qi) independently extracted the following parameters from each study: (1) first author and year of the publication; (2) patients characteristics, study design, and following-up; (3) clinical outcomes. Discrepancies between the two reviewers were resolved by discussion. The quality of all selected articles was ranked in accordance with Jadad score.

### Inclusion and exclusion criteria

Inclusion criteria for this study were as follows:(1) compare the initial therapeutic effects of RFA with or without TACE and SR for the treatment of early HCC, despite the etiology of liver disease, differences in viral hepatitis, or cirrhotic status; (2) report at least one of the outcomes mentioned below; (3) clearly document indications for RFA and HR; (4) If two or more studies were reported by the same authors in the same institution, either the study of higher quality or the most recent publication was included in the analysis. The primary endpoints were overall survival rates at 1, 3, and 5 years. The secondary endpoints were disease-free survival rates at 1, 3, and 5 years.

Criteria for exclusion: case reports, letters, abstracts, editorials, expert opinions, studies lacking control groups and reviews without original data were excluded. The following studies were also excluded: (i) those dealing with liver metastases, recurrence after hepatectomy, or unresectable HCC; (ii) those with no clearly reported outcomes of interest; (iii) those treating patients coupling with cholangiocellular carcinomas.

### Subgroup analysis

Subgroup analyses were intended to explore important clinical differences among trials that might be expected to alter the magnitude of treatment effect. A subgroup analysis was performed in this meta-analysis to consider HCC with single nodules of diameter ≤3 cm.

### Statistical analysis

We expressed results for dichotomous outcomes as odds ratio (OR) with 95% confidence interval (CI) and continuous outcome as weighted mean difference (WMD) or standard mean difference (SMD). Heterogeneity was explored by *χ*^2^ and *I*^2^. If the result of the heterogeneity test was *P* > 0.1 and *I*^2^ < 50%, ORs were pooled using the fixed-effect model(Mantel-Haenszel), otherwise, the random-effect model (DerSimonian and Laird) was used. The significance of the pooled ORs was determined by Z-test. *P* < 0.05 was considered significant.

Publication bias was assessed by visual inspection of funnel plots, in which the standard error of log(OR) of each study was plotted against its log(OR). An asymmetric plot indicates a possible publication bias. The symmetry of the funnel plot was further evaluated by Begg’s and Egger’s test. Statistical analysis was undertaken using the Stata software (version11: StataCorp, Texas, USA).

## Results

### Study selection and characteristics

After initial screening, 49 potentially relevant clinical trials of HCC were identified. Of these, 16 trials did not analyze the results of RFA separately from the other therapies, while 14 trials only focused on RFA. These 30 studies were excluded. Six trials were also excluded as no information concerning overall survival after three or five years was provided. A total of 13 studies (2 RCT and 11 NRCTs) [16,19-21,24-32] published between 2000 and 2011 were included.

These studies included a total of 2,535 patients: 1,233 treated with RAF and 1,302 with SR. The mean age ranged from 49.2 ± 9.9 to 69.4 ± 9.1 years. The male: female ratio in the pooled data was 2.57: 1. The median or mean tumor size (cm) ranged from 1.8 to 3.8. The median or mean duration of follow-up ranged from 22.7 to 847 months. The quality and characteristics of included studies are shown in Table 1.

**Table 1:** Quality and characteristics of included studies

**Reference**	**Date**	**Design**	**Jadad score**	**Treatment**	**Number of patients**	**Sex (M/F)**	**Mean age (years)**	**Tumor number (single/multiple)**	**Mean tumor size (cm)**
Nishikawa	2011	NRCT	1	SR	69	50/19	67.4 ± 9.7	69/0	2.68 ± 0.49
				RFA	162	95/67	68.4 ± 8.7	162/0	1.99 ± 0.62
Tashiro	2011	NRCT	1	SR	199	137/62	65.7 ± 9.0	132/67	2.1 ± 0.63
				RFA	87	53/34	66.3 ± 8.2	67/20	1.8 ± 0.52
HUNG	2011	NRCT	1	SR	229	184/45	60.07 ± 12.56	181/48	2.88 ± 1.06
				RFA	190	121/69	67.42 ± 11.45	152/38	2.37 ± 0.92
Nanashima	2010	NRCT	1	SR	144	112/32	63.6 ± 8.8	128/16	NA
				RFA	56	36/20	67.7 ± 8.5	51/5	NA
Huang	2010	RCT	4	SR	115	85/30	55.91 ± 12.68	89/26	NA
				RFA	115	79/36	56.57 ± 14.30	84/31	NA
Ueno	2009	NRCT	1	SR	123	82/41	67 (28 to 85)	110/13	2.7 ± 0.1
				RFA	155	100/55	66 (40 to 79)	101/54	2.0 ± 0.1
Guglielmi	2008	NRCT	1	SR	91	73/18	NA	69/22	NA
				RFA	109	88/21	NA	65/44	NA
Hiraoka	2008	NRCT	1	SR	59	44/15	62.4 ± 10.6	NA	22.7 ± 5.5
				RFA	105	76/29	69.4 ± 9.1	NA	19.8 ± 5.2
Abu-Hilal	2008	NRCT	3	SR	34	26/8	67	NA	3.8 (1.3 to 5)
				RFA	34	27/7	65	NA	3 (2 to 5)
Takahashi	2007	NRCT	1	SR	53	39/14	66 (41 to 80)	41/12	2.5 (1 to 5)
				RFA	171	120/51	69 (44 to 84)	124/47	2.1 (0.7 to 4.8)
Chen	2006	RCT	4	SR	90	75/15	49.4 ± 10.9	NA	NA
				RFA	71	56/15	51.9 ± 11.2	NA	NA
Hong	2005	NRCT	1	SR	93	69/24	49.2 ± 9.9	NA	2.5 ± 0.8
				RFA	55	41/14	59.1 ± 9.6	NA	2.4 ± 0.6
Vivarell	2004	NRCT	2	SR	79	57/22	65.2 ± 8.2	66/13	NA
				RFA	79	67/12	67.8 ± 8.7	46/33	NA

NA: Not available; NRCT: non-randomized controlled trial; RCT: randomized controlled trial; RFA: radiofrequency ablation; SR: surgical resection.

### Meta-analysis results

#### Overall survival rates

The meta-analysis showed that there was a significant difference in overall survival between the two groups at one year(12 trials [16,19-21,24-27,29-32], with certain heterogeneity), three years(13 trials [16,19-21,24-32], without heterogeneity) and five years(10 trials [16,24-32], without heterogeneity) and that the SR group was favored (see Table 2).

**Table 2:** Main results of the pooled data in the meta-analysis

**Variables**	**Number of references with data**	**OR (95% CI)**	**Q test (*P*value)**	***I***^**2**^**(%)**	**Z test (*P*value)**	**Begg’s test (*P*value)**	**Egger’s test (*P*value)**
Overall survival rates							
1 year	12 [16,19-21,24-27,29-32]	0.60(0.42, 0.86)	0.301	14.6	0.005	0.54	0.40
3 years	13 [16,19-21,24-32]	0.49(0.36, 0.65)	0.036	45.8	0.000	0.2	0.15
5 years	10 [16,24-32]	0.60(0.43, 0.84)	0.003	63.7	0.003	0.37	0.57
Recurrence rates							
1 year	13 [16,19-21,24-32]	1.48(1.05, 2.08)	0.001	63.4	0.025	1.00	0.61
3 years	13 [16,19-21,24-32]	1.76(1.49, 2.08)	0.000	69.9	0.000	0.20	0.15
5 years	10 [16,24-32]	1.68(1.21, 2.34)	0.02	54.4	0.002	0.86	0.92
Local recurrence	4 [16,20,24,30]	0.34(0.09, 1.28)	0.02	68.9	0.112	NA	NA
Complications	7 [16,19,24,25,27,30,32]	6.25(3.12, 12.52)	0.042	54	0.000	1	0.982

NA,not available.Q test and *I*^2^ were used to evaluate the heterogeneity of the studies; Z test was used to value the combined effect; Begg’s and Egger’s tests were used to assess the publication bias.

### Recurrence rates

Our results, as shown in Table 2, indicated that recurrence rates at one year(13 trials [16,19-21,24-32], without heterogeneity), three years(13 trials [16,19-21,24-32], without heterogeneity) and five years(10 trials [16,24-32], without heterogeneity) were significantly higher in the RFA group than in the SR group. However, no differences were found between the two groups (4 trials [16,20,24,30]) with respect to the local intrahepatic recurrence (see Table 2).

### Complications

The meta-analysis (7 trials [16,19,24,25,27,30,32] reported these data) showed that there was significant difference between the two groups (OR, 6.25 [95%CI, 3.12 to 12.52]; *P* = 0.000), without heterogeneity (see Table 2). The RFA group was favored.

### Subgroup analysis in HCCs ≤ 3 cm

**Figure 1:**
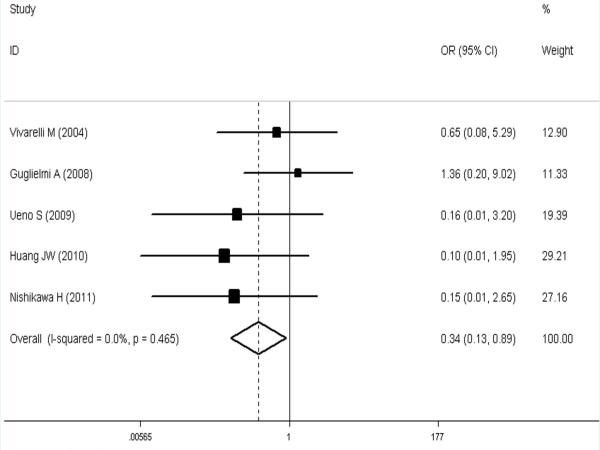
**Meta-analysis of one-year overall survival rates after SR versus RFA in HCCs ≤ 3 cm.** A fixed model was used. Pooled risk ratios are shown with 95% confidence intervals.

**Figure 2:**
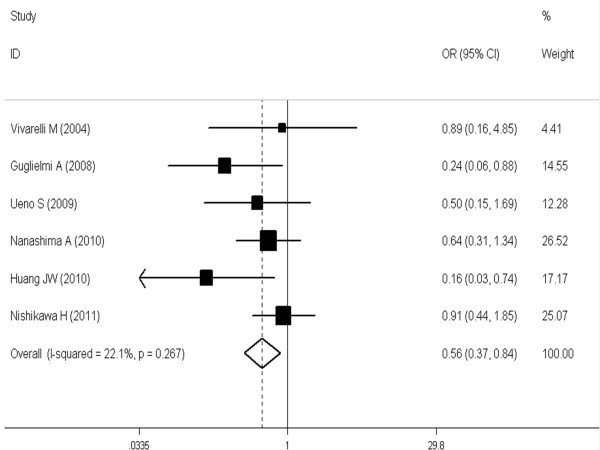
**Meta-analysis of three-year overall survival rates after SR versus RFA in HCCs ≤ 3 cm.** A fixed model was used. Pooled risk ratios are shown with 95% confidence intervals.

**Figure 3:**
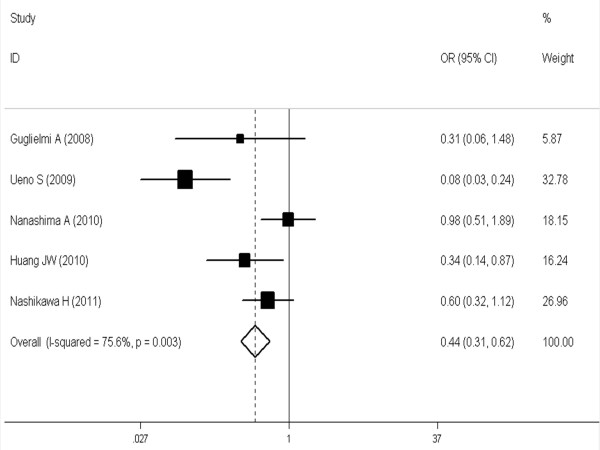
**Meta-analysis of five-year overall survival rates after SR versus RFA in HCCs ≤ 3 cm.** A fixed model was used. Pooled risk ratios are shown with 95% confidence intervals.

The meta-analysis (6 trials [16,21,24,27-29] reported these data) showed that the difference was significant and favorable to the SR group at 1, 3 and 5 years (respectively, OR, 0.34 [95%CI, 0.13 to 0.89]; OR, 0.56 [95%CI, 0.37 to 0.84]; OR, 0.44 [95%CI, 0.31 to 0.62] (see Figures 1, 2, 3).

### Sensitivity analysis

To compare the difference and evaluate the sensitivity of the meta-analysis, we employed one-way sensitivity analysis to evaluate the stability of the meta-analysis. The statistical significance of the results was not altered when any single study was omitted (data not shown). Therefore, results of the sensitivity analysis suggest that the data in this meta-analysis are relatively robust.

### Publication bias

**Figure 4:**
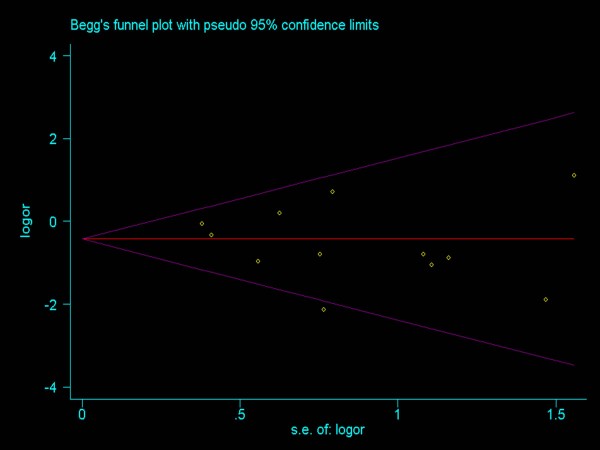
Funnel plots on one-year overall survival rates following RFA and SR for the treatment of early HCC.

Funnel plots (Figure 4) were created to assess possible publication biases. In addition, Begg’s and Egger’s tests were used to evaluate the symmetry of the plots. As shown in Table 2, the data suggest that the funnel plots were symmetrical, and that publication biases might not have an evident influence on the results of the meta-analyses.

## Discussion

There is some dispute whether survival benefits of RFA exist for patients with early HCC compared with SR. This meta-analysis demonstrated that RFA with or without TACE was inferior to SR in terms of overall survival rates and recurrence rates at one, three, and five years, contrary to the opinion of Livraghi [23]. This may be partly explained by advances in surgical and radiological techniques and perioperative care, and by more cautious patient selection [33,34]. This finding may also be adversely impacted by the delay of surveillance in effective treatment using RFA [35,36].

A high rate of recurrence after treatment is the main factor affecting overall survival and late death of patients with HCC [37]. Reportedly, the risk factors for tumor recurrence after treatment include tumor location, tumor size, multinodular tumors, and an insufficient safety margin [38-40]. Additionally, recurrences arise because of pre-existing microscopic tumor foci that were undetected by imaging modalities, or because malignant cells disseminated during operation [41,42]. In this study, recurrence was found to be more frequent after RFA than SR. This may be a result of the safety margin of RFA being narrower than that of SR, as SR usually excises the entire Couinaud segments containing tumors and possible venous tumor thrombus. In addition, high rates of recurrence after RFA may result from insufficient ablation of the primary tumor or the presence of tumor venous invasion in the adjacent liver. As for local intrahepatic recurrence rates, the two groups had no difference. This may be due to the development of techniques of RFA and an accurate evaluation of treatment response via a sufficient safety margin (at least 0.5 cm).

This meta-analysis suggested that the incidence of complications after RFA for HCC was lower than those after the SR group as a result of the microinvasive characterization of RFA. Radiofrequency ablation is a minimally invasive, target-selective technique, which has been applied in clinical studies in the 1990s [43]: it can induce thermal lesions less than 2.5 to 3.5 cm in diameter, using single expandable-tip electrodes, which are handled percutaneously and guided by imaging modalities [44]. This procedure could be performed under conscious sedation and the hospital stay is then shortened.

The subgroup analysis showed marked differences in the overall survival rates between RFA and SR for HCC ≤ 3 cm after one, three, and five years. Considering the fact that patients with single HCC ≤ 3 cm in diameter were at early stage without micro metastases and vascular invasion, SR can achieve better clinic outcomes. However, a lack of sufficient data on RCTs and an unequal constitution of patients may also affect these findings.

The majority of the data in the present study came from non-RCTs, so the overall level of clinical evidence might be low. However, a firm conclusion about bias is difficult to reach as the asymmetry of the funnel plot is minimal. Therefore our pooled OR might be an overestimate of the true effect.

## Conclusion

In conclusion, SR demonstrated significantly improved survival benefits for patients with early HCC, especially for HCC ≤ 3 cm in diameter, although local recurrence between two groups did not exhibit significant difference. However, the findings need to be carefully interpreted, owing to the lower level of evidence. Further RCTs are warranted to clarify the exact value of SR and RFA for early HCC, especially for single nodules ≤ 3 cm in diameter.

## Abbreviations

CI: confidence interval; HCC: hepatocellular carcinoma; OR: odds ratio; RCT: randomized controlled trial; RFA: radiofrequency ablation; SMD: standard mean difference; SR: surgical resection; TACE: transcatheter arterial chemoembolization; WMD: weighted mean difference.

## Competing interests

The authors declare that they have no competing interests.

## Authors’ contributions

XG, QFZ, and MY designed the studyand wrote the manuscript.XG, QFZ, ZJH, CGF, and CY performed data acquisition.MY performed quality control of data.XG, QFZ, and MY performed statistical analysis and interpretation. All authors read and approved the final manuscript. XG and QFZ contributed equally to this work.
